# Molecular epidemiology of clinically relevant single and mixed species in a Malaysian tertiary care hospital

**DOI:** 10.18502/cmm.2023.345062.1432

**Published:** 2023-06

**Authors:** Humaira Farooq, Gokul Shankar Sabesan, Tahmina Monowar, Suresh V Chinni, Noor Hasliza Zainol, SweSwe Latt, Rajesh PK

**Affiliations:** 1 Shaukat Khanum Memorial Cancer Hospital and Research Centre, Lahore, Pakistan; 2 Department of Medical Microbiology, Faculty of Medicine, AIMST University, Kedah, Malaysia; 3 Faculty of Medicine, Manipal University College Malaysia, Melaka, Malaysia; 4 Department of Microbiology, Army Medical College, Jashore, Bangladesh; 5 Department of Biochemistry, Faculty of Medicine, Bioscience, and Nursing, MAHASA University, Selangor, Malaysia; 6 Department of Periodontics, Saveetha Dental College and Hospitals, Saveetha Institute of Medical and Technical Sciences, Chennai, India; 7 Pathology Department, Hospital Sultan Abdul Halim, Ministry of Health Malaysia, Kedah, Malaysia; 8 Department of Public Health Medicine, RCSI & UCD Malaysia Campus, Penang, Malaysia; 9 Faculty of Medicine, American University of Antigua

**Keywords:** PCR-RFLP, *Msp*I, *Candida*, Mixed yeast infections

## Abstract

**Background and Purpose:**

The increasing rate of opportunistic infections caused by *Candida* and other yeasts is becoming a major health concern worldwide. However, systematic data on the epidemiology and the yeast species infections in Malaysia is still limited. In this regard, the present research aimed to identify pathogenic yeasts utilizing an economically practical and easily available molecular technique and evaluate the prevalence of pathogenic yeasts in a Malaysian tertiary care hospital.

**Materials and Methods:**

Yeast isolates were collected from Sultan Abdul Halim Hospital, Kedah, Malaysia, from October 2020 to October 2021. Molecular identification of the isolates was performed by one enzyme-based polymerase chain reaction-restriction fragment length polymorphism method.

**Results:**

*Candida albicans* was the most prevalent species, accounting for 120 isolates (59%) in total. The most prevalent non-*albicans Candida* species were *C. tropicalis* (n=33, 16%), *C. krusei* (Pichia kudriavzevii) (n=12, 5.8%), *C. glabrata* (n=12, 5.8%), and *C. parapsilosis* (n=6, 3%). Other unusual *Candida* species were *C. guilliermondii* (2), *C. metapsilosis* (2), *C. orthopsilosis* (1), *C. lusitaniae* (1), *C. rugosa* (1), *C. haemulonii* (1), *C. bracarensis* (1), and *C. dubliniensis* (1). Moreover, *Talaromyces marneffei* (1), *Kodamaea ohmeri* (1), *Cryptococcus neoformans* (3), and *Cryptococcus laurentii* (1) were among the other yeasts identified.

**Conclusion:**

The Molecular technique used in this study identified 96% of isolates, including mixed species. According to the findings, the most prevalent species are *C. albicans*, *C. tropicalis*, *C. krusei*, and *C. glabrata*.

## Introduction

The *Candida* genus includes around 154 species, six of which are most frequently isolated in human infections, namely *C. tropicalis*, *C. glabrata*, *C. parapsilosis*, *C. krusei* (*Pichia kudriavzevii*), and *C. lusitaniae*. However, the diversity of species seen in infections is growing, and additional formerly uncommon species are now more likely to arise [ 1 ]. For example, *C. auris*, a multidrug-resistant pathogen, has recently become known worldwide [ 2 ].

Every year, more than 250,000 people are affected by invasive candidiasis, with a death rate of more than 70% [ 3 , 4 ]. According to the statistics, candiduria kills patients who are hospitalized in the intensive care unit (ICU) more than other patients [ 5 - 10 ]. In many regions of the world, including Malaysia, the real epidemiology of *Candida*-based infections, their distribution, and management at the species level are missing.

Identification of infections at the species level is crucial since each species has a specific antifungal drug susceptibility pattern [ 11 ]. Another compelling reason to identify at the species level is the recent emergence of *C. auris* and other emerging species. *Candida auris* is a multi-drug-resistant pathogen and appears to be transferred from infected or colonized patients in healthcare settings; unlike most other species, *C. auris* has caused several significant outbreaks in hospitals [ 11 , 12 ]. Therefore, as the presence of *C. auris* at any place poses a risk of transmission, it is crucial to identify the species to conduct infection control measures when needed.

Therefore, we must explore methods that can be more sensitive and specific and that can rapidly and accurately identify fungal infections up to the species level. Traditional methods of identification are time-consuming and frequently result in incorrect identification. The molecular approach to diagnosis is fast becoming the accepted standard for many bacterial and viral infections, especially for the identification of isolates growing in pure cultures. However, despite being around for more than a decade, molecular tests are still not utilized as a diagnostic tool to identify fungal pathogens.

There is no known molecular method that can be applied universally and still be economically feasible. This problem also makes it difficult to find remedies that are acceptable to the Food and Drug Administration (FDA). A global shortage of medical mycologists and research in this sector limits molecular diagnostics for fungal diseases.

The current study aimed to identify yeast isolates up to the species level using an economically feasible and generally available molecular approach and find out the prevalence of common pathogenic yeasts in a tertiary care hospital in northern Malaysia. 

## Materials and Methods

### Study design and sampling

This cross-sectional study was performed using a convenience sampling technique. The sample size was calculated using the following formula: n=[DEFF*Np(1-p)]/[(d2/Z2 1-α/2*(N-1)+p*(1-p)]. Culture plates of all yeasts, including *Candida* species, were collected from the Pathology Department of Sultan Abdul Halim Hospital, Kedah, Malaysia, from October 2020 to October 2021. The samples were further processed for experiments, and the results were analyzed in the Microbiology and Molecular Biology Laboratories of the university. All positive yeast cultures, which were received from October 2020 to 2021 in the pathology lab, were included in the study, except molds.

Yeast isolates were collected from different clinical specimens (urine, blood, high vaginal swabs, and tracheal aspirates) in Sultan Abdul Halim Hospital, from October 2020 to October 2021. The Medical Research Ethics Committee of AIMST University, Kedah, Malaysia, and the Medical Research and Ethics Committee, Ministry of Health of Malaysia, approved this study under the code ID NMRR-20-1588-53243 (IIR).

### Identification of yeasts

The isolates collected from the hospital were subcultured on Sabouraud dextrose agar (Merck, Germany) and incubated at 30 °C for 48 h. Subsequently, the cultures were saved in 80% glycerol stocks at -80°. In this study, five standard strains, *Candida* (ATCC 10231), *C. parapsilosis* (ATCC 90018), *C. glabrata* (ATCC 15126), *C. tropicalis* (ATCC 1369), and *C. auris* (CDC B11903), were used as quality control.

Initially, the yeasts were identified using microscopy and *Candida* Differential Agar Media (Himedia) [ 13 ]. After initial identification, the isolates were subjected to the molecular method of polymerase chain reaction-restriction fragment length polymorphism (PCR-RFLP) [ 14 , 15 ].

The yeast DNA was extracted using the MasterPure Yeast DNA Purification Kit (Lucigen, USA). Cells were lysed non-enzymatically at 65 °C, followed by protein precipitation, nucleic acid precipitation, and DNA resuspension. The PCR-RFLP was performed based on a standard method described by Mirhendi et al. [ 14 , 15 ], with a few modifications. The referenced study used universal primers ITS1 (5-TCCGTAGGTGAACCTGCGG-3), and ITS4 (5-TCCTCCGCTTATTGATATGC-3) purchased from Integrated DNA Technologies in Singapore to amplify the ITS1- 5.8S rRNA ITS2 regions. 

The final PCR volume was 50 µL. Each reaction comprised 25 µL of 2 X PCR master mixes (Green Taq Mix, Vazyme), 1 µL of each primer (0.2 µM), 3 µL of DNA template, and 20 µL of nucleotide-free water. Conditions for PCR were as follows: initial denaturation at 95 °C for 3 min, followed by 35 cycles of denaturation for 30 s, annealing at 56 °C for 45 s, and extension for 1 min, with a final extension at 72 °C for 5 min [ 14 , 15 ]. The amplified PCR products were digested with FastDigest *Msp*I (Thermo Fisher Scientific, USA): 10 µL PCR product, 17 µL nuclease-free water, 2 µL 10× buffer, and 1 µL enzyme were mixed and incubated at 37 °C for 30 min. The PCR and RFLP products were separated on a 1.5% agarose gel in Tris-borate-EDTA buffer for 45 min at 100 V and visualized by staining with ethidium bromide.

### Interpretation of restriction fragment length polymorphism results

For molecular identification of different yeast species, the already described PCR-RFLP profiles were used [ 14 , 15 ]. The identification was made by comparing the RFLP product band pattern with reference band patterns and sizes published by the author of the method [ 15 , 16 ]. The band sizes were also confirmed by the standard curve of the ladder using the formula in Microsoft Excel.

The reference sizes of the PCR and RFLP products for the various species are displayed in Table 1. Five reference strains were also subjected to PCR-RFLP to determine whether the profiles described in the reference method were similar to those obtained on an experimental basis.

**Table 1:** Size of ITS1–ITS4 products for *Candida* species before and after digestion with *Msp*I

Yeast species	Size of PCR products (bp)	Size of RFLP digestion products (bp)
*Candida albicans*	535	297, 238
*Candida tropicalis*	524	340, 184
*Candida glabrata*	871	557, 314
*Candida parapsilosis*	530	530
*Candida guilliermondii*	607	82, 155, 370
*Candida metapsilosis*	531	531
*Candida orthopsilosis*	510	510
*Candida rugosa*	399	121, 278
*Candida lusitaniae*	382	118,264
*Candida haemulonii*	400	400
*Candida auris*	400	400
*Candida bracarensis*	805	253, 552
*Candida dubliniensis*	537	239, 298
*Cryptococcus neoformans*	555	127, 428

*Table is taken from the reference [ 16 ]

PCR polymerase chain reaction, RFLP restriction fragment length polymorphism

### Services availed for the identification of unknown species

### Sequencing

The services of 'Apical Scientific, Malaysia' were used for sequencing the unknown species. They used Sanger sequencing with the ABI PRISM 3730xl Genetic Analyzer, developed by Applied Biosystems, USA. The chemistry that they use for sequencing purposes is the BigDye® Terminator v3.1 Cycle Sequencing Kit. Furthermore, regarding the protocol of sequencing, it is a standard protocol used for preparing the sample for sequencing.

### Matrix-assisted laser desorption ionization–time-of-flight (Bruker)

To confirm the identification of a few species, the matrix-assisted laser desorption ionization–time-of-flight (MALDI-TOF) services provided by Sultan Abdul Halim Hospital were used. A portion of the colony of the microbe was placed on the sample target and overlapped with the matrix. In biotyping, the mass spectra of expressed proteins are analyzed by dedicated software and compared with stored profiles to determine species.

### Statistical analysis

Statistical analysis was performed using SPSS and Excel software. The collected data were documented in Microsoft Excel (Microsoft Corp., USA) before being transferred to SPSS (version 20, IBM, USA) for analysis. Furthermore, descriptive statistics were used to determine the frequencies of the species distributions. The relationship between species and specimen sources was found using the Chi-square test.

## Results

The hospital received 206 yeast isolates, which were recovered from 10 distinct types of clinical specimens from October 2020 to October 2021. Most of the yeast isolates were isolated from urine (n=104). Other isolates were isolated from blood (52), high vaginal swabs (45), ear discharge (2), tracheal aspirate (2), tissue (2), skin (1), nail (1), sputum (1), and cerebrospinal fluid (1), respectively.

**Figure 1:**
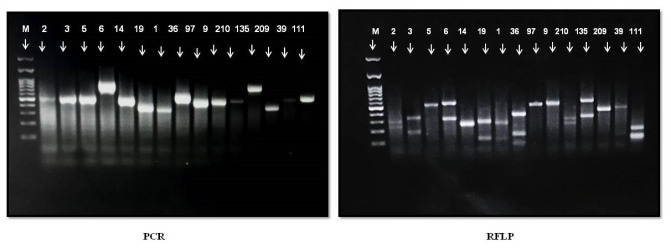
Patterns of polymerase chain reaction and restriction fragment length polymorphism products of isolates before and after digestion with restriction enzyme *Msp*I. Lane numbers represent the yeast isolate number. Lanes M, 2, 3, 5, 6, 14, 19, 1, 36, 97, 9, 210, 135, 209, 39, 40, and 111 represent DNA ladder, *Candida*, *C. tropicalis*, *C. parapsilosis*, *C. glabrata*, *C. krusei*, *C. lusitaniae*, *C. rugosa*, *C. guilliermondii*, *C. metapsilosis*, *C. orthopsilosis*, *C. dubliniensis*, *C. bracarensis*, *Kodamaea ohmeri*, *Cryptococcus neoformans*, *Cryptococcus laurentii* and *Penicillium marneffei*, respectively.

**Figure 2:**
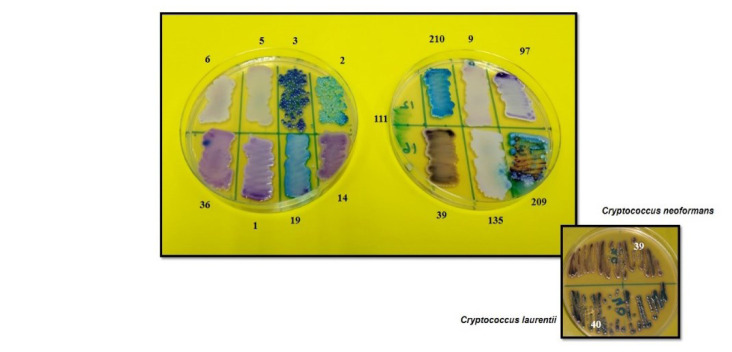
Colony color of representative isolates of different yeast species on chromogenic agar media. Numbers 2, 3, 5, 6, 14, 19, 1, 36, 97, 9, 210, 209, 135, 39, 40, and 111 represent., *Candida tropicalis*, *C. parapsilosis*, *C. glabrata*, *C. krusei*, *C. lusitaniae*, *C. rugosa*, *C. guilliermondii*, *C. metapsilosis*, *C. orthopsilosis*, *C. dubliniensis*, *Kodamaea ohmeri*, *C. bracarensis*, *Cryptococcus neoformans*, *Cryptococcus laurentii* and *Penicillium marneffei*, respectively.

The methods used in this study successfully identified all yeast isolates. The ITS-PCR-RFLP band patterns for representative yeast species identified in this study are shown in Figure 1. The fragment lengths matched the estimated sizes in the reference profile exactly. The color of the colonies for the various yeast species on the *Candida* Differential Agar is shown in Figure 2.

**Figure 3:**
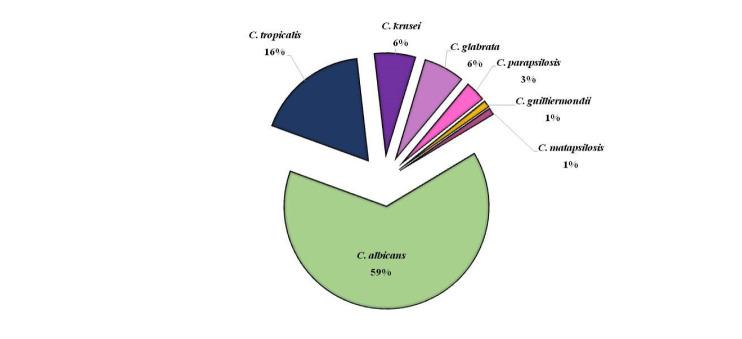
Type and frequency of common isolates

*Candida albicans* was the most prevalent species, accounting for 120 isolates (59%). The prevalence of the most common non-albicans *Candida* species was *C. tropicalis* (n=33, 16%), *C. krusei* (n=12, 5.8%), *C. glabrata* (n=12, 5.8%), and *C. parapsilosis* (n=6, 3%), respectively (Figure 3). Other uncommon *Candida* included *C. guilliermondii* (n=2), *C. metapsilosis* (n=2), *C. orthopsilosis* (n=1), *C. lusitaniae* (n=1), *C. rugosa*(n=1), *C. haemulonii*(n=1), *C. bracarensis* (n=1), and *C. dubliniensis* (n=1). In addition, *Talaromyces marneffei* (n=1), *Kodamaea ohmeri* (n=1), *Cryptococcus neoformans* (n=3), and *Cryptococcus laurentii* (n=1) were among the other yeasts identified.

Six isolates were identified as mixed yeasts (MY) species. The list and identification results of MY are summarized in Table 2.

**Table 2:** Results of mixed yeast (MY) isolates

Sr no.	Yeast 1D	RFLP
**1**	MY 1 (sample H-23)	*C. albicans*+ *C. glabrata*
**2**	MY 2 (sample H-48)	*C. albicans*+ *C. glabrata*
**3**	MY 1 (sample H-60)	*C. albicans*+ *C. glabrata*
**4**	MY 1 (sample H-63)	*C. glabrata*+ *C. tropicalis*
**5**	MY 1 (sample H-129)	*C. parapsilosis*+ *C. tropicalis*
**6**	MY 1 (sample H-177)	*C. glabrata*+ *C. albicans*+ *C. tropicalis*

Tables 3 and 4 tabulate the relationship between the distribution of different species and different types of clinical samples. Results of chi-square tests revealed that there is a significant relationship between *Candida* species and sources of specimen collection. Most of the *Candida* species were found in the urine specimen, X^2^ (15, N=206) =46.5 (*P*<0.001).

**Table 3:** Yeast species distribution with respect to specimen sources

*Candida* species	Blood	HVS	Urine	Other	Total
***C. albicans***	19	38	60	3	120
***C. tropicalis***	12	1	19	1	33
***C. glabrata***	4	1	6	1	12
***C. krusei***	1	1	10	0	12
***C. parapsilosis***	2	0	3	1	6
**Others**	11	2	6	4	23
**Total**	49	43	104	10	206

HVS: high vaginal swabs

**Table 4:** Relationship between different species and different types of clinical specimen

*Candida* species	Blood		HVS		Other		Urine		Chi-square test and *P*-value
	n	%	n	%	n	%	n	%	
*C. albicans*	19	15.8	38	31.7	3	2.5	60	50	46.5, *P*<0.001
*C. tropicalis*	12	36.4	1	3	1	3	19	57.6	
*C. glabrata*	4	33.3	1	8.3	1	8.3	6	50	
*C. krusei*	1	8.3	1	8.3	0	0	10	83.3	
*C. parapsilosis*	2	33.3	0	0	1	16.7	3	50	
Other	11	47.8	2	8.7	4	17.4	6	26.1	

HVS: high vaginal swabs

This molecular method alone could not tell the difference between *C. albicans* and *C. dubliniensis*, nor could it tell the difference between *C. parapsilosis* complex species (*C. parapsilosis*, *C. metapsilosis*, and *C. orthopsilosis*). The MALDI-TOF and sequencing methods were used to confirm these species.

The identification of *T. marneffei* and *K. ohmeri* was also confirmed by MALDI-TOF and sequencing. Even though these two species have shown a specific PCR and RFLP band pattern (Figure 1), this pattern has not been previously reported and therefore requires additional validation.

According to the prevalence formula, the prevalence of yeast infections in Sultan Abdul Halim Hospital for a certain year was 0.4%.

## Discussion

This study is the first molecular epidemiologic investigation of the species distribution profile of clinically relevant species in the northern part of Malaysia.

The distribution of *C. albicans* in this investigation is consistent with previous research. However, the trend of non-*albicans Candida* species in this study differs from those of the previous research. In the present study, *C. tropicalis* was the second most common species, followed by *C. glabrata* and *C. parapsilosis* in the third and fourth places, respectively. In most other studies, *C. parapsilosis* and *C. glabrata* were the second and third most prevalent non-*albicans Candida* infections [ 6 , 17 - 20 ].

In an Iranian study, a similar pattern of *C. tropicalis* was found [ 15 ]. *Candida krusei* was discovered at the same frequency (second) as *C. tropicalis* in the present study. However, in previous studies, it was consistently ranked fifth in frequency [ 6 , 15 , 17 - 20 ]. We also identified two unusual and rare yeast species, namely *T. marneffei* and *K. ohmeri*. 

One isolate was identified as T. marneffei, which was extracted from a blood sample of an HIV patient. *Talaromyces marneffei* is a dimorphic opportunistic fungus mostly found in Southeast Asian nations and causes widespread, life-threatening illnesses. Very little published data on this yeast is available in Malaysia. Only two case reports from Malaysia have reported this infection [ 21 , 22 ].

One isolate was identified as *K. ohmeri* in the current study, isolated from the blood of a COVID-19 patient. It is also a life-threatening illness that is seldom encountered. The first case of *K. ohmeri* fungemia was reported in the United States in 1998; however, since then, only 12 instances have been recorded there. At the same time, only three documented cases have been reported from various locations in Malaysia [ 23 - 25 ]. A significant concern in all three cases was that the patients suffered from poorly controlled diabetes mellitus. However, this is the first reported case of *K. ohmeri* in COVID-19 patients in Malaysia with no Diabetes history. The patient could not survive and died after 1 month of treatment in the ICU.

We also identified six yeast cultures, a mixture of species, with the molecular method used in this study. Mixed yeasts can go undetected by routine identification methods. Misidentifications and underreporting of cases can be the cause of such fatal situations. The rate of mixed yeast infections and frequent species combinations in this study was similar to those of previous studies from other parts of the world. In most investigations, the rate of MY infections was around 3-4%, with *C. albicans* and *C. glabrata* being the most prevalent combinations [ 26 - 29 ].

*Candida* species are frequently cultured out of urinary samples. Candiduria a common clinical finding, especially in hospitalized patients. In most situations, especially in adults, candiduria reflects colonization; however, this determination requires additional information regarding symptoms and clinical context. Risk factors for *Candida* infections in the urinary tract include female gender, extremes of age, diabetes mellitus, long-term hospitalization, ICU admission, recent use of broad-spectrum antibiotics or immune-suppressants, dysfunction of the bladder, urinary stasis, nephrolithiasis, transplantation, congenital or structural abnormalities of the urinary tract, and catheterization [ 30 ].

The proposed "one enzyme PCR-RFLP" molecular method utilized in this work has the potential to detect almost all clinically significant yeasts as well as mixed culture infections. After acquiring culture on plates, it took 6 h to report the findings. Furthermore, this method does not require buying expensive equipment or media. It can be implemented in any diagnostic lab or hospital that already has a PCR setup.

## Conclusion

Based on the results, the most common *Candida* species are *C. albicans*, *C. tropicalis*, *C. krusei*, and *C. glabrata*. This Molecular technique correctly identified 96% of the isolates tested, including mixed species and other rare species. As a result, it is possible to conclude that the PCR-RFLP technique used in this study can be a useful tool in routine laboratories for the identification of fungal infections.
